# Insights into the molecular mechanism of RGL2-mediated inhibition of seed germination in *Arabidopsis thaliana*

**DOI:** 10.1186/1471-2229-12-179

**Published:** 2012-10-04

**Authors:** Petra Stamm, Pratibha Ravindran, Bijayalaxmi Mohanty, Ee Ling Tan, Hao Yu, Prakash P Kumar

**Affiliations:** 1 Department of Biological Sciences, Faculty of Science, National University of Singapore, Singapore, 117543, Singapore; 2 Department of Chemical and Biomolecular Engineering, National University of Singapore, Singapore, 117576, Singapore; 3 Temasek Life Sciences Laboratory, National University of Singapore, 1 Research Link, Singapore, 117604, Singapore

**Keywords:** RGL2, Seed germination, Dormancy, Gibberellins, DELLAs, *Arabidopsis* microarray

## Abstract

**Background:**

Seed germination is of immense significance for agriculture and has been studied for centuries. Yet, our understanding of the molecular mechanisms underlying regulation of dormancy and germination is still in its infancy. Gibberellins are the key phytohormones that promote germination, and the DELLA protein RGL2 is the main signalling intermediate involved in this response. Germination is completely inhibited if functional RGL2 is overexpressed and/or stabilized; however, the molecular mechanisms of RGL2 function are still largely unknown. We therefore attempted to shed light onto some of the genetic events downstream of RGL2.

**Results:**

Gene ontology of the transcriptome differentially regulated by RGL2, as well as extensive cross-comparison with other available microarray data indicates that RGL2-mediated inhibition of germination causes seeds to enter a state of dormancy. RGL2 also appears to differentially regulate a number of transcription factors, many of which are known to be involved in light- or phytohormone-mediated aspects of germination. A promoter analysis of differentially expressed genes identified an enrichment of several motifs that can be bound by specific transcription factors, for example GAMYB, ARF1, or Dof-type zinc fingers. We show that Dof-binding motifs indeed play a role in RGL2-mediated transcription. Using Chromatin Immunoprecipitation (ChIP), we show that RGL2 directly downregulates at least one cell wall modifying enzyme, which is predicted to constrain cell growth thereby leading to inhibition of seed germination.

**Conclusions:**

Our results reveal that RGL2 controls various aspects of germination. Through the repression of cell wall modifying enzymes, cell growth is directly constrained to inhibit germination. Furthermore, RGL2 likely interacts with various types of proteins to regulate transcription, and differentially regulates several transcription factors. Collectively, our data indicate that gibberellins, acting via RGL2, control several aspects of seed germination.

## Background

Gibberellins are phytohormones regulating growth and development throughout a plant’s life cycle; they are essential in processes such as stem elongation, floral development and seed germination [1-4]. The importance of gibberellins in these processes is most obvious in gibberellin-deficient mutants; *GIBBERELLIC ACID REQUIRING 1 (GA1)* encodes for the *ent*-kaurene synthetase A of the gibberellin biosynthetic pathway, *ga1* plants are therefore unable to synthesise gibberellins. These mutants are extremely dwarfed, male-sterile, and most importantly their seeds fail to germinate without exogenous gibberellin [5,6].

Significant progress has been made in recent years in elucidating the gibberellin signalling pathway [7,8]. The gibberellin signal is perceived by the soluble receptors GIBBERELLIN INSENSITIVE DWARF 1 (OsGID1 or OsGID1-like) [9,10]. *Arabidopsis* contains three GID1-like genes, *GID1a*, *GID1b* and *GID1c*[11]. The gibberellin-GID1 interaction triggers the degradation of DELLA proteins, the major negative regulators of gibberellin signalling, via the 26S proteasome pathway [12-15]. The gibberellin-specific F-box proteins OsGID2 and SLEEPY1 (AtSLY1) mediate this degradation [16-19].

DELLA proteins are a subfamily of the GRAS family of transcriptional regulators [20], named after their highly conserved N-terminal DELLA motif, which mediates gibberellin-responsiveness [21-23]. In *Arabidopsis*, there are five DELLA proteins: GIBBERELLIN INSENSITIVE (GAI), REPRESSOR OF *ga1-3* (RGA), RGA-like1 (RGL1), RGL2 and RGL3 [13,24-26]. Extensive genetic studies using various combinations of DELLA knock-out mutations have elucidated overlapping as well as distinct functions of each protein in repressing plant growth and development: RGA and GAI are the main repressors of stem elongation, whereas floral development is regulated by a combination of RGA, RGL1 and RGL2, and RGL2 is the key DELLA protein repressing seed germination [24,27-31].

However, the events downstream of DELLAs in the gibberellin-mediated regulation of growth and development are less well understood. Until recently, it was not clear how DELLA proteins repress gibberellin-mediated gene expression. Although they have been classified as transcriptional regulators, they do not have conserved DNA binding domains. A major break-through in understanding the molecular mechanism of DELLA-action was achieved when the direct interaction of DELLA proteins with PHYTOCHROME INTERACTING FACTOR 3 (PIF3) and PIF4 was shown [32,33]. Since then, the main mode of DELLAs in regulating transcription is thought to occur via the sequestering of transcription factors. Recently, the interaction in yeast with more members of the basic helix-loop-helix (bHLH) subfamily 15, namely PIF3-like 5 (PIL5), PIL2 and SPATULA (SPT) was shown; the authors thus hypothesised that DELLAs could interact with all members of this subfamily [34]. This further corroborates the conclusion that the main molecular mechanism of DELLA function is their interaction with transcription factors, which leads to the formation of inactive complexes [35]. This model has been further revised to show that DELLA proteins are also able to activate transcription by sequestration of inhibitors [36-38].

Here, we focus on gibberellin-mediated regulation of germination, in particular the molecular mechanism by which the DELLA protein RGL2 suppresses germination. Germination is a complex process of three phases, each being tightly regulated at various levels. Phase I describes the intake of water, during which the seed imbibes, whereas in phase II metabolic processes are re-initiated (also called germination *sensu stricto*), and in phase III the radicle emerges [39,40]. Seed germination is regulated by the balance of the two phytohormones abscisic acid and gibberellins, which inhibit and promote germination, respectively. Gibberellins function in late phase II of germination [41], a phase which is physiologically characterised mainly by post-imbibitional cell elongation in embryonic radicle and hypocotyl as well as endosperm weakening [42]. Gibberellins are therefore key players in the actual commitment of seeds to germination.

RGL2 is the major DELLA protein involved in repressing germination [25,31]. It performs this function, at least partly, through increasing abscisic acid biosynthesis as well as activities of ABI5 and ABI3 [43,44]. This has been further elucidated by Lee *et al.*[45], showing that RGL2 regulates abscisic acid release in the endosperm to control embryo growth. In fact, RGL2 has been identified as one of the genes to be involved in the regulation of seed germination at the phase II to phase III transition [46]. Despite all these findings, it remains unclear exactly how RGL2 suppresses this complex process of germination. Therefore, elucidation of the molecular mechanism of RGL2 function would not only allow us to gain a deeper understanding of gibberellin-mediated germination, but also enable us to manipulate details of germination, for example to prevent pre-harvest sprouting in crops, at the same time ensure full and synchronous germination upon sowing.

In this study, we show that RGL2 function causes seeds to enter a state of dormancy. Microarray analysis showed that RGL2 up-regulates several genes associated with dormant states of seeds. Enforcing dormancy is partly achieved by RGL2 directly inhibiting transcription of cell wall-modulating genes *ALPHA EXPANSIN3* (*EXPA3*) and *EXPA8*, by binding to their promoters, perhaps as a complex with as yet unidentified transcription factors. Thus, RGL2 directly affects cell growth. These data suggest that RGL2 inhibits seed germination both directly and indirectly.

## Results

### RGL2-mediated transcriptome in non-germinating seeds

**Figure 1:**
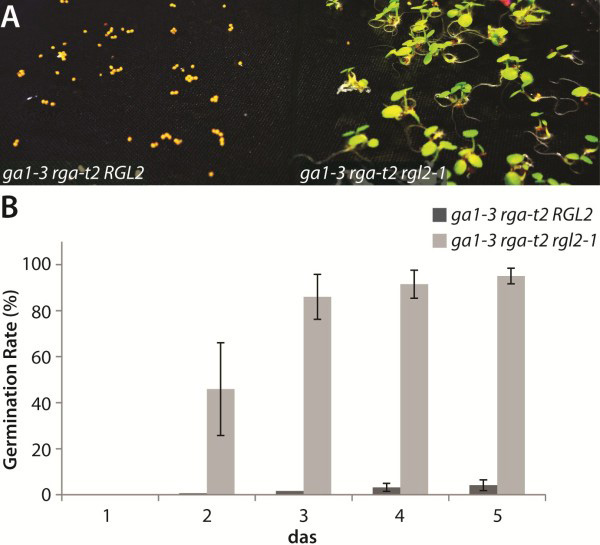
**A mutation in RGL2 can rescue the non-germinating phenotype of the gibberellin-deficient mutant.** Seeds of the double mutant *ga1-3 rga-t2* fail to germinate, due to stabilised RGL2, whereas the mutation of RGL2 in the triple mutant *ga1-3 rga-t2 rgl2-1* rescues germination to near wild-type levels. Germination rate was determined over a period of five days following stratification of seeds at 4°C for five days. Error bars indicated SD of three biological replicates. Seeds were deemed germinated when the radicle had visibly emerged from the seed coat. Pictures are representative of seeds/seedlings five days after imbibition.

In order to identify genes that are differentially regulated by RGL2 to repress germination, an oligonucleotide-based DNA microarray analysis (Agilent 60-mer gene expression microarray, 4x44k) was performed. We compared the transcriptome of the *ga1-3 rga-t2* mutant seeds, which are unable to germinate, with that from *ga1-3 rga-t2 rgl2-1*, in which germination is rescued to near wild type levels (Figure 1). Our aim was to identify target genes specifically regulated by RGL2. We therefore chose seeds from both gibberellin-deficient as well as *RGA* knock-out backgrounds. With the gibberellin-deficient background we aimed to exclude gibberellin-mediated, DELLA-independent target genes [47]. The knock-out of *RGA* in both genotypes was meant to further narrow our results down to RGL2-specific targets, since RGA plays a minor, additive role in repressing seed germination [31]. RNA was obtained from seeds after imbibition for five days at 4°C. This cold treatment promotes as well as synchronises germination [41,48]. Hence, the RNA isolated from seeds treated in such a way should represent the steady-state transcriptomes of seeds poised for germination. Two microarray replicates were performed for each genotype with independently obtained RNA. We applied stringent analysis criteria, referring to genes as being RGL2-up-regulated (RGL2-UP) or RGL2-down-regulated (RGL2-DOWN), only if in both hybridisations the signal ratio of *ga1-3 rga-t2* to *ga1-3 rga-t2 rgl2-1* was equal to or more than 2-fold different, with a p-value cut-off at 0.01. Using these criteria, we identified 607 genes as putative RGL2-regulated genes, 253 being up-regulated (RGL2-UP) and 355 being down-regulated (RGL2-DOWN) (Additional file 1). 

To further understand events during the RGL2-mediated arrest in germination, and to eliminate secondary effects of the germination arrest, we classified our gene lists by gene ontology (GO) ( http://www.arabidopsis.org/tools/bulk/go/index.jsp and http://bioinfo.cau.edu.cn/agriGO/index.php) (Additional file 2). In our data, 350 and 249 of down- and up-regulated genes, respectively, were annotated. As expected, transcripts related to post-embryonic morphogenesis were significantly (p<0.01) enriched in RGL2-DOWN. In accordance with that, GO terms related to cellular components ‘plasma membrane’ and ‘plant-type cell wall’ were significantly enriched. We further found a significant over-representation of the molecular function terms ‘hydrolase activity’, ‘carboxylesterase activity’ (p<0.05) and ‘serine-type carboxypeptidase activity’ (p<0.01). The predominant functional themes in RGL2-UP were related to responses to various exogenous and endogenous cues; GO terms ‘response to oxidative stress’, ‘response to abiotic stimulus’, ‘toxin catabolic processes’ and ‘response to gibberellin’ were significantly enriched (p<0.01). With respect to molecular functions, we found a significant (p<0.01) enrichment of genes assigned ‘glutathione transferase activity’ and ‘UDP-glucosyl transferase activity’. Furthermore, 33 genes (13%) were found in the ‘transcription factor activity’ category. These include several uncharacterised transcription factors containing MYB, basic helix-loop-helix (bHLH) or AP2 domains, as well as the Homeodomain-Leucine-zipper (HD-Zip) genes *ATHB4* and *ATHB7*, *MYB111*, *WUSCHEL-related homeobox 2* (*WOX2*) and *WRKY40*, among others. Furthermore, several hormone pathways seem to be affected; genes involved in the response to salicylic acid and gibberellins are significantly enriched in the up-regulated genes, whereas the response to auxin appears as an enriched term in down-regulated genes. Also, many of the up-regulated transcription factors appear to be either responsive to or modulate responses to the phytohormones abscisic acid, ethylene, salicylic acid, brassinosteroids and gibberellins.

### RGL2-mediated transcriptome overlaps with transcriptomes of dormant wild-type seeds

Since germination is a complex process mediated by a vast number of cues, a large number of differentially regulated genes likely represent secondary responses due to the arrest of germination, rather than a direct effect of RGL2 function. We therefore aimed to narrow down our gene lists by performing cross-comparisons with other available microarray data related to seeds, germination, gibberellins and DELLA proteins. One data set, generated by Cao *et al.*[47], contains gene expression profiles of germinating seeds and developing flowers, respectively, of wild type cv. Landsberg *erecta* (L*er*), *ga1-3*, and the *ga1-3 rga gai rgl1 rgl2* quintuple mutant, identifying both DELLA-dependent as well as DELLA-independent or partially dependent genes. Another study identified direct target genes of RGA in seedling shoots [49]. We also compared our data with the list of differentially regulated genes in seeds of the *comatose* (*cts-1*) mutant, compared to dormant (D) and after-ripened (AR) seeds of wild type cv. Landsberg *erecta* (L*er*) [50]. *COMATOSE* (*CTS*) encodes for a peroxisomal ATP binding cassette transporter, which is required for seedling establishment and survival just before radicle protrusion, downstream of RGL2; thus, *cts-1* mutant seeds remain “forever dormant”. Lastly, *PHYTOCHROME INTERACTING FACTOR 3-LIKE5* (*PIL5*), encoding for a bHLH transcription factor, is a key negative regulator of germination [51], and directly interacts with DELLA proteins [34]. Therefore, we also compared our data set with direct target genes of PIL5 that were identified by both Chromatin Immunoprecipitation-chip (ChIP-chip) and microarray data [52].

**Figure 2:**
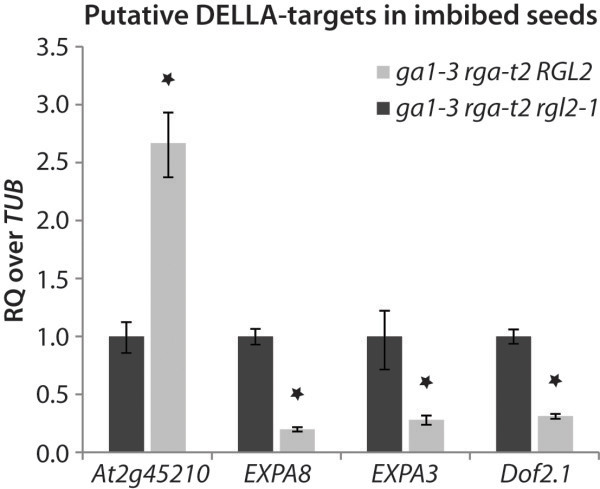
**Relative expression levels of known DELLA target genes in seeds.** Seeds were stratified in water, and RNA was extracted after five days. Expression levels of known DELLA-targets were determined by qRT-PCR in imbibed seeds of *ga1-3 rga*, relative to *Tubulin (TUB*), and compared to *ga1-3 rga rgl2-1*. Asterisks indicate a significant difference in expression levels (p<0.05). RQ = relative quantity of transcript.

Despite various differences between these data sets, most importantly the experimental set-up (Affymetrix ATH1 vs. Agilent 4x44k array), we observed a considerable overlap of gene expression profiles (Additional file 3). Of our RGL2-UP list, 89 genes (35%) overlap with at least one of these data sets, 58 of which are up-regulated in dormant seeds as identified by Carrera *et al.*[50], whereas 146 genes are unique for our data. Likewise, 177 genes (50%) of RGL2-DOWN genes were identified in at least one other data set, 110 of which are down-regulated in dormant seeds [50], with 177 genes being unique for our experiment. This not only validates the quality of our data, but also indicates the physiological state in which seeds appear to be arrested due to RGL2 action. Twelve of the genes that have been identified as direct target genes of PIL5 [52] can also be found in our data (six genes in RGL2-UP, six in RGL2-DOWN). In total, we found 75 and 17 down- and up-regulated genes, respectively, that were identified as DELLA-dependent transcripts in seeds [47]. Interestingly, some of these (three genes in RGL2-UP, six genes in RGL2-DOWN) appear to be DELLA targets in both flower buds and seeds. Thus, these genes likely represent direct targets of RGL2. Lastly, three of the genes directly targeted by RGA in seedling shoots [49] can also be found in our data, including *GIBBERELLIN 20-OXIDASE* (*GA20ox*) and *GIBBERELLIN INSENSITIVE DWARF1b* (*GID1b*). We therefore determined the expression levels of some of the genes that are likely direct targets of RGL2 in imbibed seeds of *ga1-3 rga-t2* and *ga1-3 rga-t2 rgl2-1* (Figure 2). The as yet uncharacterised gene *At2g45210* of the *SMALL AUXIN UPREGULATED RNA* (*SAUR*)-family, which appears to be a DELLA target gene in both flowers and seeds, is expressed at much higher levels in imbibed seeds of the *ga1-3 rga-t2* mutant, indicating that it could be up-regulated by RGL2. We also confirmed the expression of the PIL5 target gene *ALPHA EXPANSIN 8* (*AtEXPA8*) and the related *AtEXPA3*, as well as the DNA-binding with one finger (Dof)-type transcription factor *Dof2.1*, all of which are down-regulated in the presence of RGL2. The observed expression levels are consistent with our microarray data, which further supports the hypothesis that these genes could be directly regulated by RGL2. 

### Several cis-elements are abundant in promoters of RGL2-regulated genes

We further analysed our data, aiming to understand how transcriptional regulation by RGL2 is coordinated. For this, we identified motifs that are over-represented in the promoter regions of differentially regulated genes. Promoter regions (−1,000 to +200) of the RGL2-regulated genes, were used for *ab initio* detection of putative cis-elements using the Dragon Motif Builder algorithms. Motifs with occurrences of 20% or higher were matched with known promoter elements in the TRANSFAC, PLACE and AGRIS databases. This way, we identified 14 motifs in promoters of up-regulated (Table 1) and nine in promoters of down-regulated genes (Table 2). In promoters of both RGL2-UP and RGL2-DOWN genes, gibberellin response element (GARE)-like motifs, which are associated with GAMYB-type transcription factors, are the most significantly enriched motifs. Furthermore, motifs associated with the auxin signalling intermediate *AUXIN RESPONSE FACTOR1* (*ARF1*) are significantly enriched in promoters of up-regulated genes, with three out of the 14 motifs detected belonging to this category. Interestingly, three out of the 14 motifs detected in RGL2-UP are associated with transcription factors of the Dof-type (‘DNA-binding with one finger’), two of which (AAAAG element and TAAAG element) are present in more than 35% of the promoters analysed.

**Table 1:** Cis-elements enriched in promoters of RGL2-up-regulated genes

**Consensus sequence**	**Putative cis-element**^**1**^	**Associated type of transcription factor**	**%**^**2**^	**TIC**^**3**^	**e-value**
TTTTTCAA	Pyrimidine box-like / PE1/AT-hook element-like / GT element-like	GAMYB / PF1, GT1 / GT3b	46	14.53	2e-005
AAAAAAAAAG	AAAAG element / PE1/AT-hook element-like	Dof1, Dof4, Dof11, Dof16 / PF1	42	19.02	0e+000
AATAAAGA	TAAAG element	Dof4, Dof11, Dof16	37	14.50	0e+000
TCTCTCTTT	GAGA element-like / TCA-1 binding site	GAGA-binding factor BBR / TCA-1	32	17.00	2e-005
TATTTGTTT	GARE-like / AuxRE-like	GAMYB / ARF1	31	16.08	0e+000
TTAGTTTT	Myb box-like	MYB1	29	15.03	2e-005
TTTGTTTTC	Pyrimidine box-like/GARE / GT element-like / AuxRE-like	GAMYB / HD PR2 / ARF1	27	16.23	0e+000
TTTCTTTG	TCA-1 binding site	TCA-1 (tobacco nuclear protein 1)	26	16.00	0e+000
TGCTTCTC	CAMTA3 binding site / AuxRE-like / IDE1 (iron-deficiency-responsive element 1)	CAMTA3 / ARF1	26	13.44	3e-005
AAAAAAATG	AT hook/PE1 element-like	PF1 / DBP	23	16.45	2e-005
CTTATAT	TATA-box	TBP	21	15.10	0e+000
CAAGAATG	AAGAA motif	Dof2	20	14.03	0e+000
GATTTTGTT	GARE-like	GAMYB	20	16.05	3e-005
AAGACAAA	GARE-like	GAMYB	20	16.00	0e+000

^1^Homology with motifs identified in *Arabidopsis* and other plant species (TRANSFAC, PLACE and AGRIS databases).

^2^Percent occurrence in critical promoters relative to background sequences.

^3^Total Information Content.

Promoter motifs identified in promoters of RGL2-UP, with consensus sequence, name and associated transcription factors.

**Table 2:** Cis-elements enriched in promoters of RGL2-down-regulated genes

**Consensus sequence**	**Putative of cis-element**^**1**^	**Associated type of transcription factor**	**%**^**2**^	**TIC**^**3**^	**e-value**
TCCAAAAA	AT-hook/PE1 element-like / L-box/UV-B responsive element-like	PF1	38	14.52	2e-005
GTTTTTTTT	GARE-like / AT-hook/PE1 element-like	GAMYB / PF1	34	18.00	0e+000
TATAACAA	MYB box-like / GARE-like	GAMYB	33	14.51	0e+000
TTTATTTTA	Box III-like	GT-1	32	17.13	2e-005
AAATTTCA	GT element-like	GT-1	30	15.01	0e+000
TTTCTTTG	TCA-1 (tobacco nuclear protein 1) binding site / T-box-like	TCA-1	23	16.00	2e-005
AGAAAGTG	phyA-induced motifs / Sucrose Responsive Element (SURE)-like		23	15.00	0e+000
AATTATTTA	TATA-box	TBP	21	16.58	2e-005
ATTATGAA	JASE1/JASE2-like	AP2/ERF (jasmonate inducible)	20	15.00	2e-005

^1^Homology with motifs identified in Arabidopsis and other plant species (TRANSFAC, PLACE and AGRIS databases).

^2^Percent occurrence in critical promoters relative to background sequences.

^3^Total Information Content.

Promoter motifs identified in promoters of RGL2-DOWN, with consensus sequence, name and associated transcription factors.

**Figure 3:**
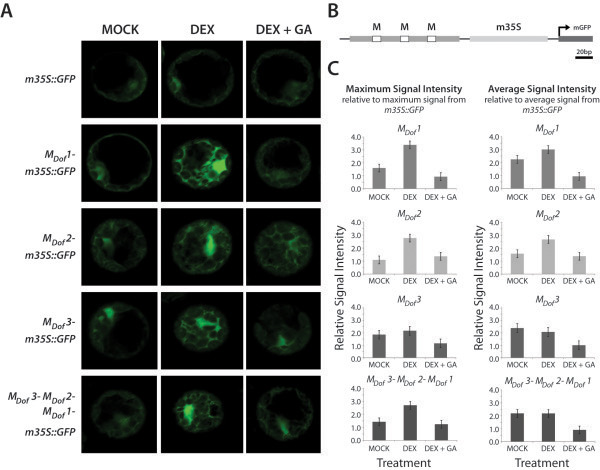
**Transcriptional activation by RGL2 involves promoter motifs bound by Dof-type transcription factors.** Mesophyll protoplasts were transfected with reporter constructs carrying different promoter motifs 5’ of a minimal promoter (*m35S*) to drive GFP expression (**B**). Transfected protoplasts were treated with MOCK, DEX, or DEX plus GA_3_, and GFP signal was analysed by confocal microscopy (**A**). Signal intensities of 6 to 10 protoplasts of each construct/treatment-combination were determined using the Carl Zeiss LSM software, and plotted relative to the signal obtained from GFP driven by *m35S* only (**C**).

**Figure 4:**
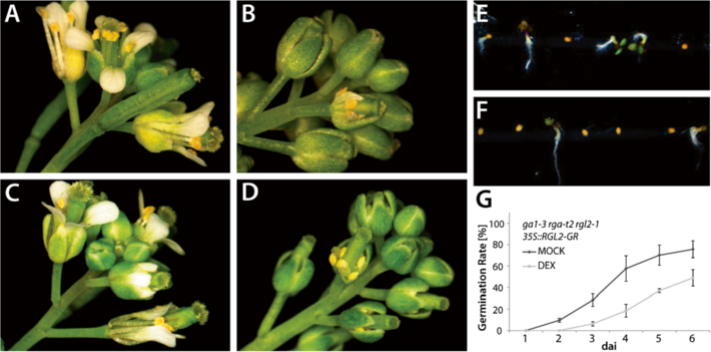
**RGL2 tagged with GR is biologically functional.** Flower development and seed germination phenotype of *ga1-3 rga-t2 rgl2-1 35S::RGL2-GR* without (**A**, **E**) and with (**B**, **F**) DEX-treatment. Without DEX-treatment, RGL2-GR fusion protein is held in the cytoplasm, thus unable to perform its function; the flower phenotype therefore resembles the triple mutant *ga1-3 rga-t2 rgl2-1* (**C**), and seeds readily germinate upon imbibition (**E**, **G**). Upon DEX-treatment, RGL2-GR can enter the nucleus and regulate transcription; the flower phenotype is reverted to the double mutant *ga1-3 rga-t2* phenotype with sterile flowers with stunted petals and stamens (**D**), and germination is significantly delayed and inhibited (**F**, **G**). Two sepals of one flower each have been removed in (**B**) and (**D**) to reveal the short petals and stamens. Germination was scored in three biological replicates. Error bars indicate SD. dai – days after imbibition.

To further evaluate the biological significance of our *ab initio* promoter motif prediction, we tested the Dof-recognised motifs for binding by the RGL2-complex *in vivo*. These motifs will henceforth be referred to as M_Dof_1 (AAAAG element), M_Dof_2 (TAAAG element) and M_Dof_3 (AAGAA element). We generated reporter constructs containing three tandem copies of the consensus sequence of each motif 5’ of the minimal 35S promoter to drive the expression of GFP (Figure 3B) and transfected *Arabidopsis* mesophyll protoplasts derived from a *35S::RGL2-GR ga1-3 rga-t2 rgl2-1* transgenic line. These plants constitutively express *RGL2* fused to the rat glucocorticoid-receptor, which allows for inducible nuclear translocation by treatment with dexamethasone (DEX). This system was described as a ‘potent tool in examining transcriptional activation’ and has been successfully used before [30,53]. Without treatment, plants of this transgenic line bear no difference to those of the triple mutant *ga1-3 rga-t2 rgl2-1*. Upon DEX-treatment, RGL2-GR fusion protein can enter the nucleus, which in the intact plants reverts both flower development and seed germination to the gibberellin-deficient phenotype (to resemble the double mutant *ga1-3 rga-t2*), indicating that the fusion protein is functional (Figure 4). After transfection, protoplasts were either treated with (1) 0.01% ethanol (MOCK), (2) DEX in 0.01% ethanol, to induce RGL2 translocation into the nucleus, or (3) DEX plus gibberellic acid (GA_3_), which will promote the degradation of RGL2. Thus, if the selected promoter elements are involved in RGL2-mediated transcriptional regulation, we expected to observe changes in GFP-signal intensity; upon DEX-treatment, GFP-signal should increase compared to that observed in MOCK-treated transfected protoplasts, whereas protoplasts treated with DEX plus GA_3_ should not exhibit different intensities of GFP. Indeed, we were able to observe a significant increase in GFP-intensity in protoplasts containing constructs with two of the three Dof-binding motifs tested (Figure 3A), both with respect to maximum as well as average intensity of fluorescence within a given protoplast (Figure 3C). This increase of GFP-intensity indicates a transcriptional activation by RGL2 (as a complex with other proteins) that is dependent on the presence of these Dof-binding motifs. Interestingly, highest GFP-induction was observed with M_Dof_1, which occurs at the highest frequency (42%) in our data. Collectively, these results indicate that the predicted motifs in our analysis are indeed cis-regulatory elements involved in RGL2-mediated regulation of germination. 

### RGL2 directly regulates several genes affecting various physiological aspects to inhibit germination

**Figure 5:**
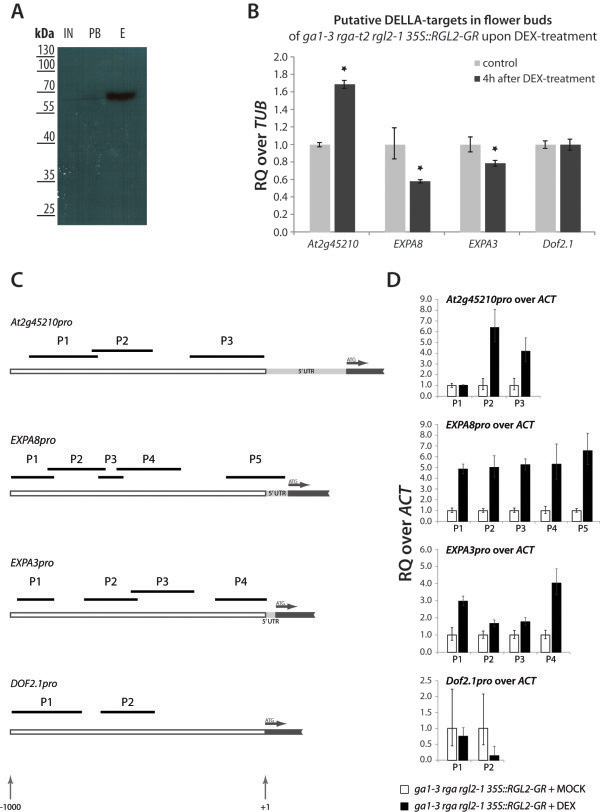
**RGL2-GR directly binds to the promoters of various genes.** (**A**) Western Blot using anti-GR antibody on samples at various stages of the protein-chromatin isolation; IN, input, PB, post binding, E, eluted chromatin/protein complex. (**B**) Relative expression of putative RGL2-/DELLA-target genes in flower buds of *ga1-3 rga-t2 rgl2-1 35S::RGL2-GR* 4h after DEX-treatment, relative to *Tubulin* (*TUB*). Asterisks indicate a significant difference in expression levels compared to the control (p<0.05). (**C**) ChIP-qRT-PCR analysis of selected target gene promoters. Promoter regions from +1,000 to +1 relative to the transcription start site are depicted, and the position of fragments amplified by qRT-PCR is given as black bars on top. Graphs indicate the relative enrichment of these fragments over *ACTIN2* (*ACT2*) in chromatin isolated from *ga1-3 rga-t2 rgl2-1 35S::RGL2-GR*, 3h after Dex-treatment, compared to chromatin from Mock-treated flower buds. Data were obtained from biological triplicates. RQ = relative quantity.

To confirm if a selected few genes from our microarray dataset are directly regulated by RGL2, we performed a Chromatin Immunoprecipitation (ChIP)-quantitative real-time PCR (qRT-PCR) analysis using the *35S::RGL2-GR ga1-3 rga-t2 rgl2-1* transgenic line. Chromatin fragments were isolated from flower buds of 4- to 5-week-old plants 3h after treatment with 10μM DEX, using monoclonal anti-GR antibodies. Chromatin isolated in the same manner from Mock-treated flower buds served as control. Since DELLA proteins do not directly bind to DNA, we used two steps of cross-linking during tissue fixation. First, we performed a protein-protein cross-linking using disuccinimidyl glutarate (DSG) [54], after which we cross-linked protein with DNA using formaldehyde, followed by chromatin isolation. We tested the efficiency of our protein pull-down by Western Blot, on which we observed a single band for GR-tagged RGL2 (Figure 5A). This not only indicates that we were able to specifically pull down RGL2, but also highlights the efficiency of our additional cross-linking, since no signal from RGL2-GR can be detected post binding. As a further control for our experimental set-up, we isolated RNA from flower bud tissue 4h after DEX-treatment, and determined expression levels of putative RGL2 target genes identified from our microarray analysis (Figure 5B). Overall, the expression changes of the putative target genes tested overlap with those observed in seeds earlier. However, the changes in expression levels appear to be more moderate compared to those seen in imbibed seeds, with *Dof2.1* not showing any change in expression upon DEX-treatment. 

For each putative RGL2-target promoter, we designed primers to amplify approximately 90 to 250 bp fragments within the region 1,000 bp upstream of the transcription start site (TSS) (Figure 5C). In isolated chromatin, we determined the enrichment of those promoter fragments over *ACTIN2* (*ACT2*), relative to the control, by qRT-PCR. Overall, we observed a 4- to 6.5-fold enrichment of specific promoter fragments (Figure 5C). From the promoters of downregulated genes tested, both *EXPA3* and *EXPA8* appear to be bound by the complex involving RGL2; however, the promoter of *EXPA8* shows a significantly higher enrichment of all fragments tested, compared to *EXPA3*. Promoter fragments of the Dof-type transcription factor *Dof2.1* did not show any significant enrichment, which correlates with the lack of transcriptional response in this tissue (Figure 5B). Among promoters of up-regulated genes, the promoter of *At2g45210*, an as yet uncharacterised gene of the SMALL AUXIN UPREGULATED RNA (SAUR)-protein family, shows a strong enrichment.

### Selected RGL2-target genes play a role in the regulation of germination in response to gibberellin and abscisic acid

We selected *At2g45210*, *ATHB2* and *ATHB5* as representative RGL2 target genes in an attempt to further validate our microarray data. *At2g45210* is a member of the SAUR-gene family of unknown function. It appears as a DELLA-target in both flowers and seeds, and our ChIP data indicate that it is a direct target gene of RGL2. *ATHB2* and *ATHB5* were chosen as representative homeobox transcription factors that are likely downstream of RGL2 function. *ATHB2* appears as up-regulated gene in our microarray only at lower stringency (p<0.05), but the expression change in seeds of *ga1-3 rga-t2 rgl2-1* vs. *ga1-3 rga-t2* could be confirmed (Additional file 4). On the other hand, *ATHB5* appears to be down-regulated in seeds with stabilised RGL2. Both genes have been characterised previously, with ATHB2 being one of the key transcription factors involved in the regulation of shade avoidance [55], and ATHB5 playing a role in the abscisic acid-mediated repression of germination and seedling root growth [56]. However, no data are available regarding the regulation of germination by *ATHB2*, or the molecular mechanism by which *ATHB5* increases abscisic acid sensitivity in seeds. We were therefore intrigued to explore the possibility of their involvement in gibberellin-mediated germination control.

**Figure 6:**
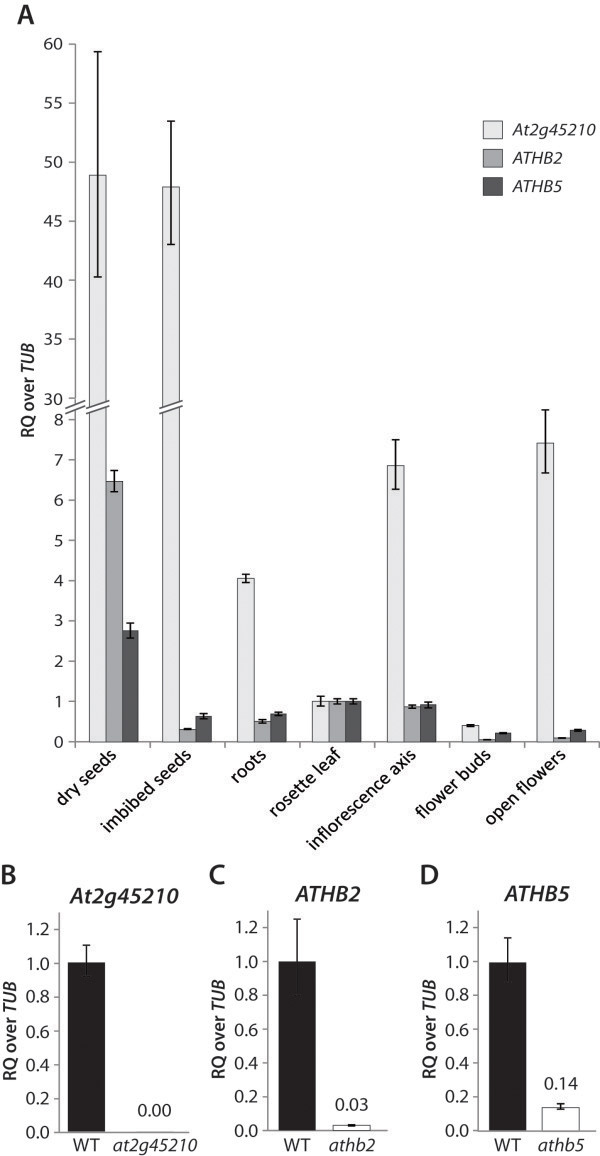
**Relative expression levels of *ATHB2*, *ATHB5 *and *At2g45210 *are high in seeds.** Relative expression levels of the RGL2 target genes *ATHB2*, *ATHB5* and *At2g45210* in various tissues of wild type plants as determined by qRT-PCR, relative to *Tubulin* (*TUB*), compared to the expression in rosette leaves (**A**). All three genes appear to be highly expressed in seed tissues, suggesting a role in the regulation of seed/embryo development or maturation. In addition, *At2g454210* is highly expressed in flowers, suggesting additional functions in flower development. (**B**-**D**) Expression levels of *ATHB2*, *ATHB5* and *At2g45210* in dry seeds of respective T-DNA insertion lines. RQ = relative quantity of transcripts.

Significantly higher transcript levels were detected for these three genes in both dry and imbibed seeds, compared to any other tissue of mature plants tested (Figure 6A), suggesting that they could indeed play a role in seed development, maturation and/or germination. Interestingly, expression of *At2g45210* is higher than that of *ATHB2* and *ATHB5* in all plant tissues tested, and showed a more than 18-fold increase from flower buds (up to developmental stage 13) to open flowers (stage 14).

**Figure 7:**
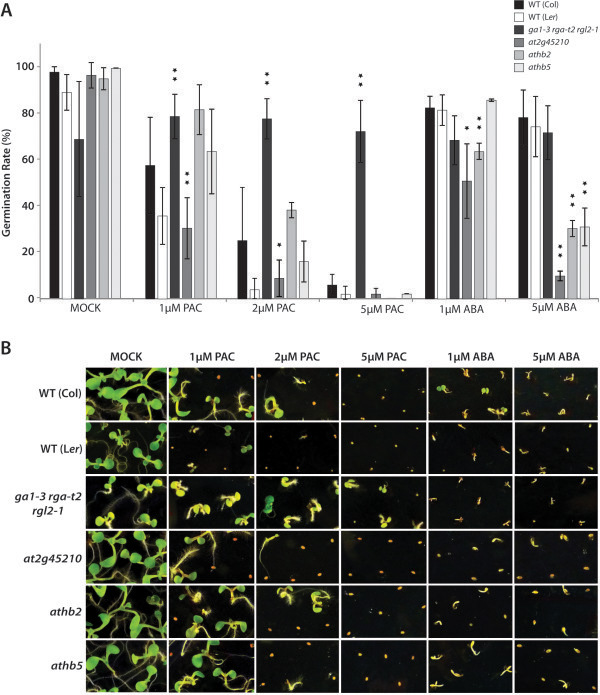
**Germination responses are affected in mutants of *ATHB2*, *ATHB5 *and *At2g45210.*** Seeds of wild type and insertion lines were surface sterilised and, without stratification, germinated on 1xMS, supplemented with 0.01% ethanol (MOCK), 1, 2 or 5 μM Paclobutrazol (PAC), 1 or 5 μM abscisic acid (ABA). Germination was scored six days after imbibition. Seeds were deemed germinated if the radicle had visibly emerged from the seed coat. Statistical differences were analysed using a two-tailed t-test, and significant differences are indicated by one and two asterisks indicating p-values of p<0.05 and p<0.01, respectively.

We obtained T-DNA insertion lines for the three genes, which showed low or no expression of the targeted gene (Figure 6B-D). Germination responses in these mutants were analysed to confirm their involvement in RGL2-, thus gibberellin-mediated germination regulation. Germination rates were examined in response to paclobutrazol (PAC), a gibberellin biosynthesis inhibitor, as well as to abscisic acid, which opposes gibberellin action in germination (Figure 7). As controls, we analysed germination responses of both wild type and the *ga1-3 rga-t2 rgl2-1* mutant. The loss of function of *RGL2* in this triple mutant does not only rescue the non-germinating phenotype of the *ga1-3 rga-t2* mutant (Figure 1), but also renders these seeds insensitive to PAC (Figure 7). However, their germination responses to abscisic acid did not differ significantly from those of wild type. Germination of *at2g45210* seeds was more severely inhibited than the wild-type seeds by all treatments, indicating that this mutant is hypersensitive to both PAC and abscisic acid with respect to germination. The effect of PAC on seed germination of *athb2* and *athb5* mutants, on the other hand, was not significantly different from that of the wild type. However, *athb2* and *athb5* mutant seeds differed in their germination response to abscisic acid; at a low concentration (1μM), germination of *athb2*, but not *athb5*, is more strongly inhibited than germination of wild-type seeds. However, at higher concentrations of abscisic acid tested, germination of both mutants is inhibited to a greater extent than that of the wild type. The increased sensitivity to abscisic acid of the *athb2* and *athb5* mutant seeds further prompted us to test whether expression of either gene is responsive to abscisic acid treatment. We therefore determined the relative expression of *ATHB2* and *ATHB5* in *ga1-3 rga-t2 rgl2-1* and *ga1-3 rga-t2* seeds that were imbibed in water or abscisic acid (Additional file 4). Interestingly, the transcript levels of the two genes do not differ between the water- and abscisic acid-treatments. In the presence of RGL2, *ATHB2* is expressed at a significantly higher level, while *ATHB5* shows lower transcript levels.

## Discussion

### RGL2-mediated arrest of germination likely induces a state of secondary dormancy

Due to its immense significance for agriculture germination is one of the most extensively studied fields of plant physiology. Yet, the molecular mechanisms underlying the regulation of germination and dormancy are still largely unknown. In this study, we aimed to elucidate some of the downstream events of the DELLA protein RGL2 in the suppression of germination. Our microarray analysis showed that in non-germinating seeds with stabilised RGL2 protein, genes associated with various stress-responses are enriched among up-regulated genes, along with gibberellin-responsive genes. This is in line with previous transcriptome analyses indicating a high representation of genes related to abscisic acid and stress responses in dormant seeds. On the other hand, as expected, genes associated with embryonic morphogenesis appear to be over-represented in down-regulated genes. More valuable information, however, could be inferred by comparing our gene lists with other available seed- and germination-related microarray data. Overall, the transcriptome of *ga1-3 rga-t2* seeds strongly resembles that of dormant seeds, suggesting that seeds with stabilised RGL2 are either unable to leave primary dormancy, or that they are entering a state of secondary dormancy upon imbibition. Thus, RGL2 could be involved in induction or maintenance of seed dormancy. However, we can only speculate if RGL2 maintains primary dormancy or induces secondary dormancy. Nonetheless, it had been determined that gibberellins, thus RGL2, act late in phase II of the germination process. At this stage seeds have already left primary dormancy, thus have committed to germination. Based on our data, we propose that RGL2 function causes seeds to enter a state of secondary dormancy, in part by directly arresting embryonic growth.

According to our data, RGL2 also seems to positively regulate the expression of a number of transcription factors of MYB-, AP2/ERF-, bHLH- and HD-Zip-types, which are involved in various responses to phytohormones as well as developmental stages. For example, *ATHB4* has previously been characterised as a regulator of shade avoidance, being strongly up-regulated by far red light, and modulating the responses to auxins, brassinosteroids and gibberellins in seedlings [57]. *MYB111* was shown to be mainly expressed in cotyledons of seedlings, and it plays a role in flavonol accumulation [58]. Two transcription factors have previously been shown to be involved in seed development or germination; *WOX2* regulates apical embryo patterning during seed development [59,60], whereas *WRKY40*, which is strongly up-regulated by abscisic acid, is a transcriptional repressor in abscisic acid and abiotic stress responses [61]. It is tempting to speculate that RGL2 controls far more aspects of seed/embryo development and germination than previously thought, since we observed up-regulation of numerous transcription factors in our microarray analysis. However, the exact transcriptional network governed by RGL2 action, and the relationship between these transcription factors and RGL2 function remains to be further elucidated.

DELLAs have been shown to physically interact with a number of bHLH-type transcription factors, leading to the hypothesis that interaction with bHLH-type of transcription factors is the main underlying molecular mechanism for DELLA function [32-34]. Importantly, it was shown that DELLAs directly interact with PIL5, an inhibitor of seed germination [52]; it is therefore not surprising to find several of its direct target genes in our data. For example, *PHYTOCHROME INTERACTING FACTOR3-LIKE2* (*PIL2*) is 3.4-fold up-regulated in our data. It belongs to a light-responsive bHLH transcription factor family, and has been shown to be involved in seed germination responses to red/far red light [62]. Interestingly, PIL2 was also shown to physically interact with RGL2 [34], indicating that RGL2 might be intricately involved in the light-dependent phytochrome-mediated regulation of germination. In RGL2-DOWN, *EXPANSIN A8* (*EXPA8*) and *CYSTEINE PROTEINASE1* (*CP1*), both more than 3.5-fold downregulated, overlap with PIL5 direct targets, amongst others. *CP1* belongs to a group of cell wall proteins involved in cell wall modulation and cell growth [63]. Expansins are a group of cell-wall modulating enzymes, many members of which have been shown to be responsive to cold-, abscisic acid- and gibberellin-treatment [reviewed in: 42]. Thus, both genes are involved in weakening cross-links of cell wall components, which allows cell elongation. Therefore, they are considered to be at the “end” of the signalling cascade that leads to cell growth. More importantly, both *EXPA8* and *CP1* have also been identified as DELLA-dependent transcripts [47], further indicating that they could be direct target genes of RGL2.

### RGL2-mediated transcriptional regulation should involve the interaction with various classes of transcription factors

To obtain a better understanding of the regulatory network underlying RGL2 function in the repression of seed germination, we performed an *ab initio* promoter motif detection in promoters of differentially regulated genes. We identified several motifs that are overrepresented in our data. In promoters of both up- and down-regulated genes, we detected a number of GAREs, motifs that are typically bound by transcription factors of the GAMYB-type. GAMYB transcription factors respond to gibberellin signalling, and activate transcription of gibberellin-responsive genes [64,65]. This suggests that our microarray data are indeed enriched for genes involved in the gibberellin-mediated regulation of germination, corroborating the quality of our data. It furthermore allows us to hypothesise that RGL2 could interact with GAMYB transcription factors to regulate transcription. Since GARE-like motifs can be found in both RGL2-UP and RGL2-DOWN, this transcriptional regulation is likely mediated by other proteins that form complexes with RGL2. Further investigations into RGL2-interacting proteins are required to clarify this. In addition, motifs associated with the auxin signalling-related transcription factor ARF1 appear to be enriched in up-regulated genes. This allows us to hypothesise that RGL2-dependent signalling pathway could also interact with auxin signalling in the regulation of embryonic growth.

Furthermore, we observed a strong enrichment of motifs associated with Dof-type transcription factors in the promoters of up-regulated transcripts. Using a protoplast assay, we could confirm that these motifs are indeed involved in the RGL2-mediated transcriptional activation of target genes *in planta*. It is therefore tempting to speculate that RGL2 might also interact with Dof transcription factors to activate transcription. However, we cannot exclude the possibility that RGL2 binds to and inhibits competitors or inhibitors of Dof proteins, thus allowing Dof proteins to bind to their target promoters and induce transcription, a similar scenario as was reported for DELLA action in the jasmonic acid signalling pathway [36]. Dof proteins are a family of plant-specific transcription factors with some 36 members in *Arabidopsis*, many of which have been implicated in the regulation of germination. For example, the Dof zinc finger protein Dof Affecting Germination1 (DAG1) and DAG2 have been shown to possess opposing roles in the regulation of germination, with DAG1 inhibiting germination by mediating PIL5 activity as well as directly affecting gibberellin biosynthesis [66,67]. Another Dof transcription factor, Dof6, was shown to negatively regulate germination by affecting abscisic acid signalling in seeds [68]. It is therefore likely that Dof proteins play crucial roles in the gibberellin-mediated regulation of germination, and that the enrichment of promoter motifs recognised by Dof transcription factors in our data is of biological significance. It also suggests that either Dof transcription factors themselves or their inhibitors could be binding partners of RGL2. It has been reported that all Dof proteins, likely due to the high similarity of their DNA binding domains, recognise similar target sequences, containing a CTTT consensus core [69]. It was therefore proposed that specificity with regards to spatiotemporal expression and/or interaction with other proteins confers specific functions to each Dof protein [67]. In seeds, it is thus possible that the expression of Dof target genes is specified to phase II of germination through RGL2-action; to shed more light on this complex process of germination, it would be of great interest to identify which specific Dof proteins are involved in the RGL2-mediated repression of germination. Generally, DELLA proteins have been reported to interact with the bHLH class of proteins [32-34] to affect transcription. Our results show that RGL2 likely interacts with various other classes of transcription factors to mediate its response.

### RGL2 regulates the expression of various genes to inhibit germination both directly and indirectly

Based on our ChIP-qRT-PCR analyses designed to further understand the molecular events downstream of RGL2, we selected a few genes that were likely direct targets. DELLA proteins lack a DNA binding domain, and are thought to regulate transcription through binding to other proteins, most of which are still unknown. We therefore employed a ‘double’ cross-linking, using DSG and formaldehyde consecutively to capture the whole protein complex bound to chromatin. Previous ChIP assays with DELLA proteins were reported to yield only subtle enrichment (1.3- to 3.5-fold) of target promoter fragments [49]; here, we were able to achieve a significantly higher enrichment (4- to 6.5-fold). This suggests that the application of DSG is a promising method of identifying chromatin regions bound by a protein complex in plants, even if only one of the proteins is known, as has been shown in human cell culture systems [54].

In our data, the RGL2-complex appears to bind to promoters of both *EXPA3* and *EXPA8,* however, promoter fragments of *EXPA8* appear more strongly enriched than those of *EXPA3*. These different levels of enrichment could be due to different binding affinities of the RGL2-complex to these promoters; it could also indicate different binding partners of RGL2 in the complex that binds to DNA. The stronger enrichment of the *EXPA8* promoter is in line with our expression analysis showing a more subtle down-regulation of *EXPA3* in flower buds of the inducible *ga1-3 rga-t2 rgl2-1 35S::RGL2-GR* mutant. Previous studies had also shown that *EXPA8* expression is more strongly gibberellin-inducible than *EXPA3*[42]. Furthermore, *EXPA8* appears to be a DELLA-target gene in both flowers and seeds [47]. We therefore propose that the RGL2-complex directly downregulates *EXPA8* expression in both flower development and seed germination; thus, RGL2 inhibits both developmental responses by directly constraining cell elongation growth.

Our data further show that the RGL2-complex appears to bind directly to the promoter of a member of the SAUR gene family, *At2g45210*, whose expression is up-regulated in our microarray data. This corroborates further the “updated” gibberellin signalling model [70], which includes not only transcriptional silencing through sequestering of transcription factors by DELLA proteins, but also transcriptional activation by sequestering inhibitors of transcription factors.

Our observation that seed germination in the loss-of-function mutant of *At2g45210* exhibits hypersensitivity to abscisic acid and PAC compared to the wild type indicates a possible role for *At2g45210* in promoting seed germination. However, its expression is up-regulated by RGL2, an inhibitor of seed germination, and was found to be high in all dormant states of seeds (primary and several secondary dormancy states) analysed by Cadman *et al.*[71]. This apparent inconsistency of results needs further investigation of this gene’s function in seed germination and dormancy. It is possible that it is not directly involved in the inhibition or promotion of seed germination *per se*. Additionally, we cannot exclude the possibility that its expression is controlled by several other factors not considered here. This is in fact a likely scenario, since its expression pattern only partly overlaps with that of *RGL2*; it is relatively high in all wild-type tissues tested, whereas *RGL2* expression is restricted to imbibed seeds and flower buds [25].

We further investigated the role in the regulation of germination of two homeobox genes, *ATHB2* and *ATHB5*, which were identified in our microarray as up- and downregulated by RGL2, respectively. Both genes have been studied with respect to their roles in other developmental stages; however, no or little data are available to determine their functions in the regulation of seed germination. Interestingly, our seed germination assays appear to reveal different roles for *ATHB2* and *ATHB5* in germination; while the inhibition of gibberellin biosynthesis with PAC does not result in a germination response different to that of wild type in either mutant, low concentrations of abscisic acid appear to inhibit seed germination more strongly than that of wild type in *athb2*, but not *athb5* . However, at high concentrations of abscisic acid, both mutant seeds appear more sensitive to its inhibitory effect. This could indicate that *ATHB2* is involved in the abscisic acid-mediated regulation of germination, independently of gibberellin. A similar conclusion could be drawn for the role of *ATHB5* in the regulation of seed germination; however, the increase in sensitivity of seed germination to abscisic acid is not as high as in *athb2*. Further germination assays using several intermediate concentrations of abscisic acid would help to determine the range of sensitivity. Interestingly, our data further indicate that expression levels of either *ATHB2* or *ATHB5* are not affected by abscisic acid treatment. This suggests that the regulation of these two genes is downstream of RGL2, but upstream of the abscisic acid signalling pathway. However, further studies need to be performed, for example, crossing the loss-of-function mutants with various mutants of abscisic acid biosynthesis or signalling, in order to place either gene in the genetic network of gibberellin and abscisic acid signalling. Nevertheless, our results indicate that both these homeobox genes affect seed germination.

## Conclusions

Taken together, our data reveal that the role of RGL2 in the inhibition of germination is complex. We show that RGL2 downregulates the expression of genes encoding cell wall modifying enzymes, viz., *CP1* and *EXPA8*, with at least *EXPA8* being directly regulated. Thus, RGL2 directly constrains cell elongation growth to inhibit germination. Our microarray data also indicate that various types of transcription factors are differentially regulated by RGL2, suggesting that RGL2 regulates several aspects of seed germination in addition to cell elongation growth, including responses to several phytohormones and light. Our promoter analysis further indicates that RGL2 interacts with various proteins to regulate transcription, including GAMYB, ARF1 and Dof transcription factors. Lastly, we investigated germination responses of three selected target genes to PAC and abscisic acid. We show that sensitivity of germination to at least one of these treatments is increased in these mutants, which not only indicates that all of these genes are in some way involved in the regulation of germination, but also validates the quality and reliability of our data. However, more work needs to be done to understand the mechanism of regulation of germination, and to shed more light on the genetic network underlying the complex process of seed germination in which RGL2 is but one player.

## Methods

### Plant materials, plant transformation and growth conditions

*Arabidopsis thaliana* accessions used in this study were either Columbia (Col-0) or Landsberg *erecta* (L*er*). Plants were grown in a growth chamber with a 16-h-light/8-h-dark cycle, at 23°C and 75% RH for generation of transgenic plants and seed collection. Plant transformation was performed as described by Clough and Bent [72] using *Agrobacterium tumefaciens* strain GV3101:pMP90.

All gibberellin-related mutants described here are in L*er* background; *ga1-3 rga-t2* and *ga1-3 rga-t2 rgl2-1* were described earlier [30]. The inducible transgenic plants *ga1-3 rga-t2 rgl2-1 35S::RGL2-GR* were generated by transforming *ga1-3 rga-t2 rgl2-1* plants with the binary vector harbouring the *35S::RGL2-GR* cassette. Insertion lines for *At2g45210* (SALK_142329), *ATHB2* (SALK_106790C) and *ATHB5* (SALK_122765) were obtained from the ABRC seed stock, and are in Columbia (Col-0) background. Homozygous plants were identified by genotyping with primers designed using the T-DNA primer design tool ( http://signal.salk.edu/tdnaprimers.2.html; Additional file 5).

For germination assays, seeds were surface sterilised in 75% ethanol and 15% commercial bleach, followed by at least five rinses with sterile water. Aseptic seeds were placed on 1xMS medium, pH 5.7 with 0.5% Gelrite, supplemented with either of the following: 0.01% ethanol (MOCK), 10μM gibberellic acid 3 (GA_3_), 1μM, 2μM or 5μM paclobutrazol (PAC), 1μM or 5μM abscisic acid, 10μM dexamethasone (DEX). To analyse germination inhibition by DEX of the inducible *ga1-3 rga-t2 rgl2-1 35S::RGL2-GR* mutant, aseptic seeds were vacuum-infiltrated with 30μM DEX prior to being placed on MS plates. All plates were incubated at 22°C with a 16-h-light/8-h-dark cycle. Germination was scored up to six days after imbibition. Seeds were deemed germinated if the radicle had visibly protruded through the seed coat.

### Microarray experiments and data analysis

Total RNA was isolated from seeds imbibed in water at 4°C in the dark for five days, according to Vicient and Delseny [73], with slight modifications. For each isolation 50mg (dry weight) seeds were used. Crude RNA was purified by extractions with chloroform, phenol, phenol:chloroform:isoamyl alcohol (25:24:1) and chloroform:isoamyl alcohol (24:1), once each. After precipitation, RNA was dissolved in 30 to 50μl water.

Total RNA was sent to Genotypic Technology [P] Ltd., Bangalore, India for microarray analysis (Project No. GT-537_E), including RNA quality control using Bioanalyser, reverse transcription and labelling, single colour hybridisation onto Agilent *Arabidopsis* 4×44k Array, preliminary data analysis with GeneSpring GX version 10.0 and Excel, data normalisation using Percentile Shift Normalisation and Normalisation to Specific Samples. Microarray data were deposited in the Gene Expression Omnibus (GEO; http://www.ncbi.nlm.nih.gov/projects/geo), accession number GSE40485.

### Chromatin immunoprecipitation

Chromatin Immunoprecipitation (ChIP) was performed using flower buds of 3-4-week-old *ga1-3 rga-t2 rgl2-1 35S::RGL2-GR* plants. Plants were sprayed with either of the following: 0.01% ethanol (MOCK), or 10μM DEX, and flower buds were harvested 3h after treatment. ChIP was performed according to Kaufmann *et al.*[74], with minor changes. We included an additional step of protein-protein cross-linking using 10mM disuccinimidyl glutarate (DSG) [54], prior to cross-linking of DNA-protein complexes by formaldehyde. All centrifugation steps were performed at maximum speed (~17,000x*g*). For the detection of RGL2-GR, total extracts, flow through and eluates prior to proteinase K treatment were resolved under reducing conditions on 12% SDS/polyacrylamide gel, and proteins were transferred onto polyvinylidene difluoride (PVDF) membranes (Bio-Rad Laboratories). PVDF membranes were blocked by incubation with 5% milk powder in Phosphate-buffered saline + 0.05% Tween 20 (PBS-T) over night, followed by incubation with monoclonal mouse anti-GR antibody (1:1,000) at room temperature for 2h. After washing with PBS-T, membranes were incubated with secondary antibody, horseradish peroxidase- (HRP-) conjugated rabbit anti-mouse antibody (1:10,000) for 2h at room temperature. Membranes were then washed four times with PBS-T, and immune complexes were detected on x-ray film (Fuji medical x-ray film) using the Enhanced Chemiluminescence (ECL) Detection kit according to the manufacturer’s instructions (Amersham Pharmacia).

### Quantitative real-time PCR (qRT-PCR)

Comparative analysis of selected genes was performed by qRT-PCR. Reactions were performed on cDNA, prepared from RNA of various *Arabidopsis* tissues using ‘MAXIMA® First Strand cDNA Synthesis Kit’ (Fermentas), with ‘KAPA SYBR® FAST qPCR Kit’ (KAPA Biosystems) using the ‘StepOne™ Real-Time PCR Systems’ (Applied Biosystems). All qRT-PCR data were generated from biological duplicates. Relative quantification of expression was determined using ‘StepOne Software’ (v2.1). For a list of primers, see Additional file 5.

### *Ab initio* promoter analysis

The entire sets of up-regulated (253) and down-regulated (354) genes, respectively, were analysed for enrichment of promoter motifs. Sequences of promoter regions (−1,000 to +200 nt relative to TSS) were extracted from our in-house promoter sequence database. Over-represented motifs were detected using the Dragon Motif Builder algorithm with EM2 option [75], and thirty motifs of 8 to 10 nucleotides were detected each with a threshold value of 0.95. Random promoter sequences were used for background subtraction of random motif occurrence. Significant motifs were selected based on a threshold occurrence of more than 20%, and motif classes were identified by significant matches with TRANSFAC [76], PLACE [77] and AGRIS [78,79] databases.

### Plasmid construction, protoplast isolation and transfection

For promoter motif analysis, a minimal promoter (90bp of CaMV 35S promoter) was amplified with SmaI and BamHI restriction sites, and cloned into pGreen (HY105 backbone) containing mGFP (cloned with SpeI and XbaI), to generate pGreen-*m35S::GFP*. Synthetic promoter constructs containing three tandem copies of selected promoter motifs were generated by overlapping PCR using synthesised oligonucleotides (see Additional file 5). The generated 126bp-fragments were cloned into the binary vector using HindIII and PstI restriction sites, 5’ of the minimal promoter in pGreen-*m35S::GFP*.

Leaf mesophyll protoplasts were isolated from 3- to 4-week-old wild-type *Arabidopsis* (Col-0) plants following the protocol described in Yoo *et al.*[80]. For each transfection, 10 to 15μg of plasmid DNA was used, and treatment occurred 30min after transfection with either of the following: 0.01% ethanol (MOCK), 10μM DEX, 10μM DEX plus 10μM GA_3_. Images were acquired four to six hours after transfection using a Carl Zeiss Axiovert 200M confocal laser microscope ( http://www.zeiss.de/axiovert200) with excitation at 488nm. For GFP detection, one channel was configured between 505 and 530nm. All images were recorded with the same detection settings. Average and maximum signal intensity of 6 to 10 selected protoplasts was determined using the Carl Zeiss LSM software (ver. 4.0).

## Abbreviations

ChIP: chromatin immunoprecipitation; DEX: dexamethasone; Dof: DNA binding with one finger; *EXPA8*: *Expansin A 8*; GA: gibberellic acid; GFP: green fluorescence protein; *RGL2*: *RGA-like 2*; qRT-PCR: quantitative real-time PCR.

## Competing interests

There are no competing interests.

## Authors’ contributions

PS designed and performed all experiments, including all statistical and bioinformatics analyses, and wrote the manuscript. PR optimised and performed RNA extractions, ChIP and qRT-PCR experiments. BM performed the *ab initio* promoter analysis. ELT generated the inducible line. HY helped in designing the project and writing the manuscript. PPK was responsible for overall supervision of experimental design, data analysis, and writing the manuscript. All authors approved the final manuscript.

## Supplementary Material

Additional file 1 **Genes differentially regulated in seeds of *ga1-3 rga-t2 *vs. *ga1-3 rga-t2 rgl2-1.*** List of genes identified as RGL2-UP and RGL2-DOWN, respectively, with a fold-change of at least 2, and a p-value ≤0.01. Click here for file

Additional file 2 **Gene Ontology (GO) analysis of the RGL2-mediated transcriptome in seeds.** GO analysis according to AgriGO (http://bioinfo.cau.edu.cn/agriGO/index.php), with respect to biological process (A, C) and molecular function (B, D) in RGL2-UP (A, B) and RGL2-DOWN (C, D). Click here for file

Additional file 3 **Cross-comparison of RGL2-mediated transcriptome in seeds with other available microarrays.** List of genes differentially regulated by RGL2 in seeds that were identified in other available microarray data sets. Overlapping genes are indicated by ‘+’. Click here for file

Additional file 4 **Relative expression levels of *ATHB2 *and *ATHB5 *in response to abscisic acid.** Seeds were stratified in water or 5μM abscisic acid, and RNA was extracted after 12h. Expression levels of *ATHB2* and *ATHB5* were determined by qRT-PCR in imbibed seeds of *ga1-3 rga*, relative to *Tubulin (TUB*), and compared to *ga1-3 rga rgl2-1*. RQ = relative quantity of transcript. Click here for file

Additional file 5 **Primers and oligonucleotides used.** List of primers and oligonucleotides used for genotyping of T-DNA insertion lines, qRT-PCR, ChIP-qRT-PCR, and for construction of reporter constructs containing different promoter motifs. Click here for file
