# Supplementary figures and images for: Insights into the molecular mechanism of RGL2-mediated inhibition of seed germination in Arabidopsis thaliana
Source: BMC Plant Biol. 2012 Oct 4;12:179. doi: 10.1186/1471-2229-12-179 (PMC3732085; doi:10.1186/1471-2229-12-179)

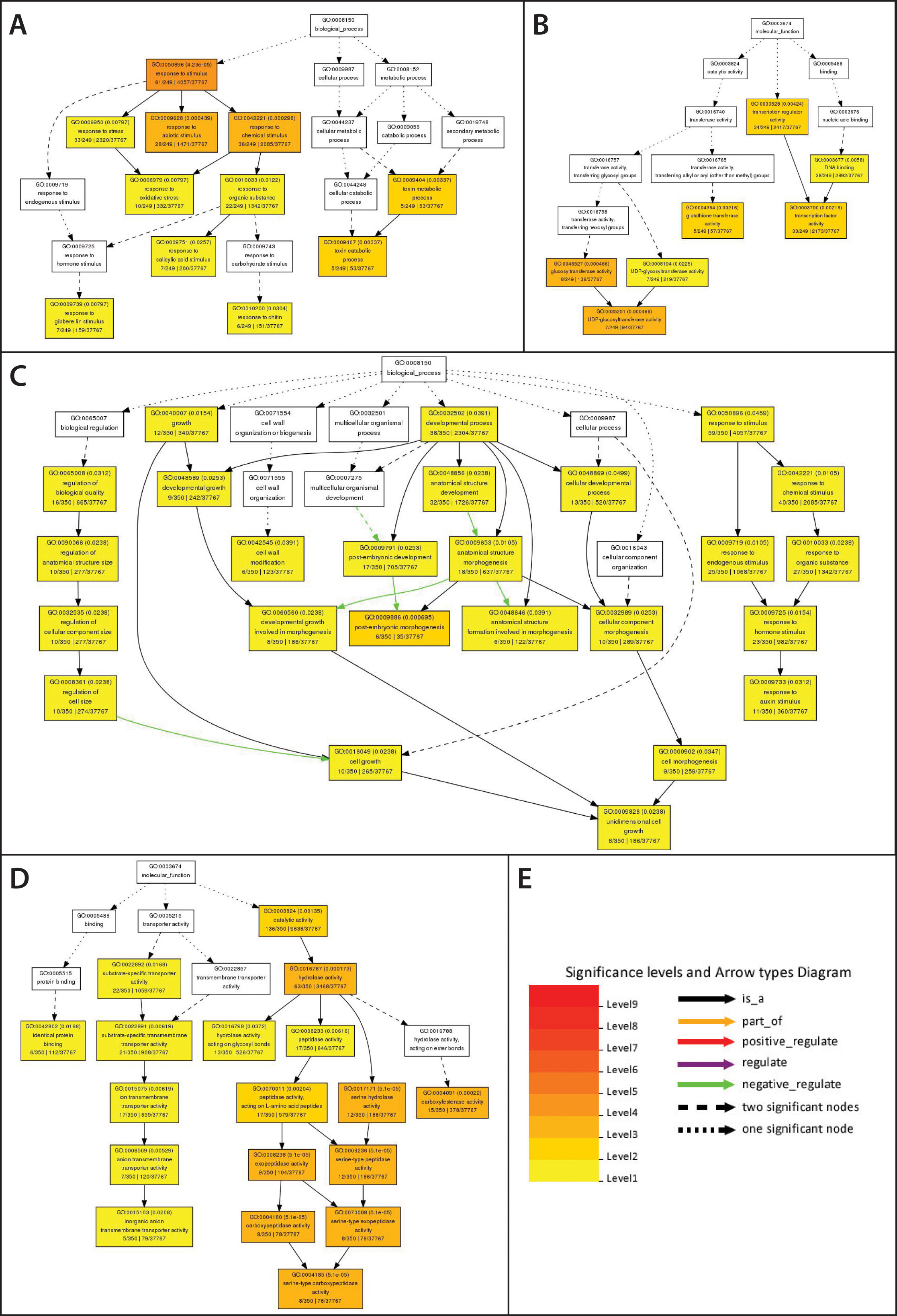

Supplement: Additional file 2 — Gene Ontology (GO) analysis of the RGL2-mediated transcriptome in seeds. GO analysis according to AgriGO (http://bioinfo.cau.edu.cn/agriGO/index.php), with respect to biological process (A, C) and molecular function (B, D) in RGL2-UP (A, B) and RGL2-DOWN (C, D). [file 1471-2229-12-179-S2.jpeg]

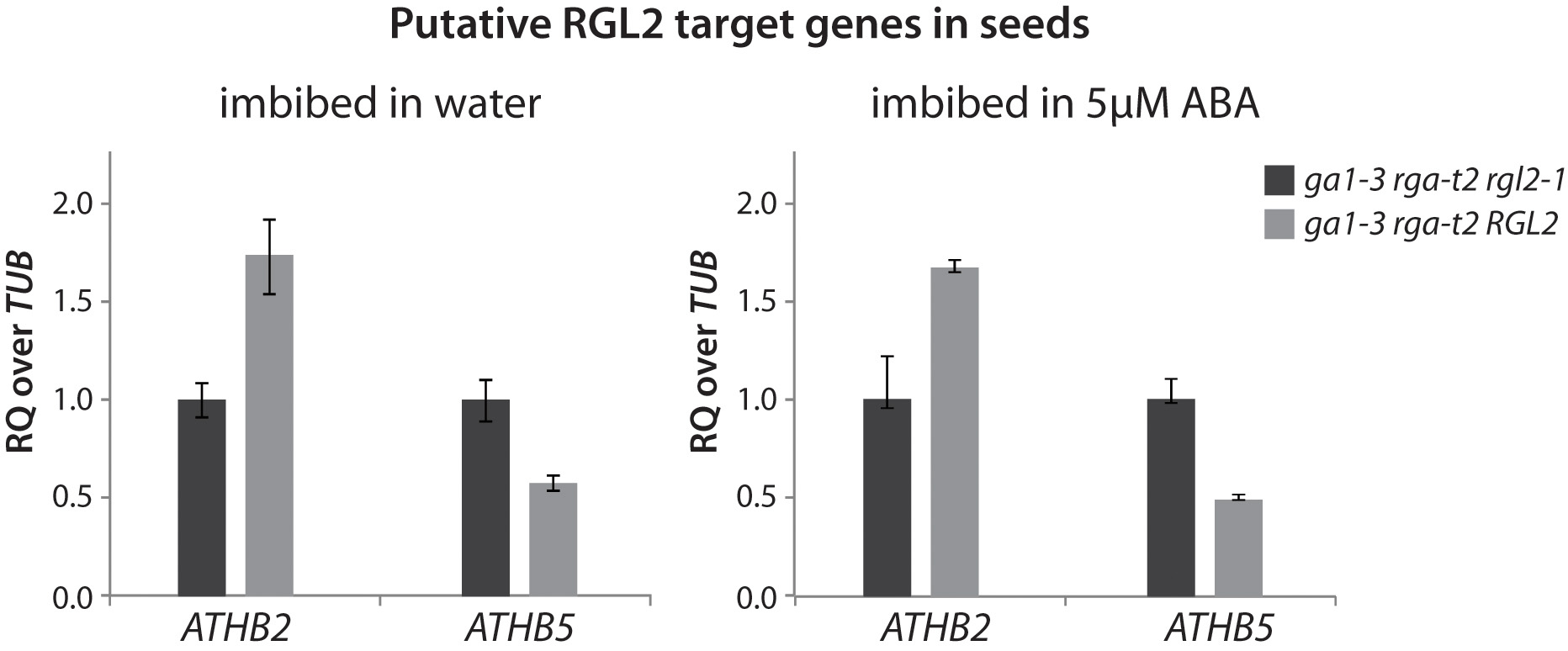

Supplement: Additional file 4 — Relative expression levels of ATHB2 and ATHB5 in response to abscisic acid. Seeds were stratified in water or 5μM abscisic acid, and RNA was extracted after 12h. Expression levels of ATHB2 and ATHB5 were determined by qRT-PCR in imbibed seeds of ga1-3 rga, relative to Tubulin (TUB), and compared to ga1-3 rga rgl2-1. RQ = relative quantity of transcript. [file 1471-2229-12-179-S4.jpeg]
